# An Account of Successful Treatment of a Case of Suppression of Urine

**Published:** 1822-10

**Authors:** Wm. Bidwell

**Affiliations:** Member of the Royal College of Surgeons, London.


ART. VI.—*An Account of successful Treatment of a Case of Suppression of Urine.* By WM. BIDWELL, Member of the Royal College of Surgeons, London.

ON Thursday, the 8th of August, I was called to a man named Jenner, a carpenter, residing at Burwash, who had not voided urine for three days. He was verging on sixty, and of a plethoric habit. He complained of great pain in the course of the ureters and in the hypogastric region; anxiety was strongly depicted on his countenance; the pulsations of the radial artery were hard, full, and jarring, not exceeding ninety. No probable cause could be assigned for the complaint. Mr. Weston, the attending practitioner, had previously abstracted twenty-four ounces of blood, administered saline cathartic medicines, recommended the warm bath, and passed the catheter. On examination, I could not perceive that intumescence above the pubes which characterizes retention; nor did the sensation of a distended bladder present itself to the finger in the rectum.

I considered it, however, my duty to pass the catheter, but no water followed its introduction. I was now convinced the case was suppression. I ordered him to be bled in the erect posture, and, though nearly three pounds were abstracted, the depletion was not attended by syncope. I directed that he should be immersed in the warm bath twice during the night, and prescribed the following:

℞. Pulv. Digital. purp. gr. j.
Syr. q. s. fiat pilula, omni 3tia horâ, cum haustu sequente sumenda.

℞. Misturæ Camph. ℥jss.

Spirit. Æth. Nit. ʒiij. M. fiat haustus.

On visiting him the next morning, he expressed considerable relief, though the renal glands had not resumed their secretory functions. Perceiving great tumefaction, I applied cupping glasses over the lumbar region, and abstracted blood to the extent of three pints. The anguish of the patient was still further alleviated by this evacuation. Convinced that nothing but bold and decisive practice could afford the most distant hopes of recovery, I ordered as follows:

℞. Hyd. Submuriatis, gr. j.
Pulv. Digitalis, gr. ij. Fiat pilula, 3tia quâque horâ cum haustu sequente sumenda.

℞. Inf. Digitalis fol. recent. ℥jss.

Spirit. Ætheris Nitr. ʒiij. Fiat haustus.

And this plan of treatment to be persisted in as long as the suppression lasted.

I now took leave, requesting Mr. Weston to inform me of any material change in the patient. On Sunday afternoon he sent word, that the urinary secretion continued suspended, and that singultus and tendency to coma had supervened, with a sinking pulse. Conceiving the case as hopeless, I ordered the digitalis to be omitted, and to take the following:

℞. Æther. Rect. ℥ss.

Misturæ Camph. ℥vj. M. Capt. cochl. ij larg. 3tia quâque horâ.

On Tuesday I was informed that the man had discharged large quantities of urine, and was rapidly recovering. The kidneys had recommenced secreting the day previous, having been in a state of total inaction for six days.

The following reasons have induced me to publish this case:

First, This disease almost universally terminates fatally.

Secondly, The proximate cause of death has been proved to be apoplexy.

Thirdly, No regular treatment of this complaint is laid down by authors.

*Warbleton, Sussex; August 26th,* 1822.

